# Combination disease‐modifying treatment in spinal muscular atrophy: A proposed classification

**DOI:** 10.1002/acn3.51889

**Published:** 2023-09-10

**Authors:** Crystal M. Proud, Eugenio Mercuri, Richard S. Finkel, Janbernd Kirschner, Darryl C. De Vivo, Francesco Muntoni, Kayoko Saito, Eduardo F. Tizzano, Isabelle Desguerre, Susana Quijano‐Roy, Kamal Benguerba, Dheeraj Raju, Eric Faulkner, Laurent Servais

**Affiliations:** ^1^ Children's Hospital of The King's Daughters Norfolk Virginia USA; ^2^ Department of Paediatric Neurology and Nemo Clinical Centre Catholic University Rome Italy; ^3^ Center for Experimental Neurotherapeutics, St. Jude Children's Research Hospital Memphis Tennessee USA; ^4^ Department of Neuropediatrics and Muscle Disorders Medical Center University of Freiburg, Faculty of Medicine Freiburg Germany; ^5^ Departments of Neurology and Pediatrics Columbia University Irving Medical Center New York New York USA; ^6^ The Dubowitz Neuromuscular Centre University College London, Great Ormond Street Institute of Child Health & Great Ormond Street Hospital London UK; ^7^ National Institute of Health Research, Great Ormond Street Hospital Biomedical Research Centre London UK; ^8^ Institute of Medical Genetics, Tokyo Women's Medical University Tokyo Japan; ^9^ Department of Clinical and Molecular Genetics Hospital Vall d'Hebron Barcelona Spain; ^10^ Hôpital Necker Enfants Malades, APHP Paris France; ^11^ Garches Neuromuscular Reference Center (GNMH) APHP Raymond Poincare University Hospital (UVSQ Paris Saclay) Garches France; ^12^ Novartis Gene Therapies Switzerland GmbH Rotkreuz Switzerland; ^13^ Novartis Gene Therapies, Inc Bannockburn Illinois USA; ^14^ Institute for Precision and Individualized Therapy, Eshelman School of Pharmacy, University of North Carolina at Chapel Hill Chapel Hill North Carolina USA; ^15^ Genomics, Biotech and Emerging Medical Technology Institute, National Association of Managed Care Physicians Richmond Virginia USA; ^16^ Department of Paediatrics MDUK Oxford Neuromuscular Centre & NIHR Oxford Biomedical Research Centre University of Oxford Oxford UK; ^17^ Department of Paediatrics, Neuromuscular Reference Center University and University Hospital of Liège Liège Belgium

## Abstract

We sought to devise a rational, systematic approach for defining/grouping *survival motor neuron*‐targeted disease‐modifying treatment (DMT) scenarios. The proposed classification is primarily based on a two‐part differentiation: initial DMT, and persistence/discontinuation of subsequent DMT(s). Treatment categories were identified: monotherapy add‐on, transient add‐on, combination with onasemnogene abeparvovec, bridging to onasemnogene abeparvovec, and switching to onasemnogene abeparvovec. We validated this approach by applying the classification to the 443 patients currently in the RESTORE registry and explored the demographics of these different groups of patients. This work forms the basis to explore the safety and efficacy profile of the different combinations of DMT in SMA.

## Introduction

*Survival motor neuron (SMN)*‐targeted, disease‐modifying treatments (DMTs) have demonstrated efficacy and safety as single therapies across many clinical trials and are now well established as standard of care for patients with spinal muscular atrophy (SMA).^1^, ^2^, ^3^, ^4^, ^5^ Despite a lack of clinical trial and real‐world data to demonstrate the clinical benefit (or risk) associated with the administration of more than one *SMN*‐targeted DMT in combination or in sequence, a clear trend exists for the use of more than one DMT in specific circumstances (e.g., initiating bridge treatment with nusinersen or risdiplam while awaiting decline of anti‐AAV9 [adeno‐associated virus serotype 9] antibody titers to a concentration that permits onasemnogene abeparvovec administration or physician or caretaker desire to switch treatment in an effort to optimize patient outcomes).^6^, ^7^, ^8^, ^9^, ^10^, ^11^, ^12^, ^13^

No systematic approach has been proposed to classify the various combination treatment scenarios for patients with SMA who receive DMTs and to facilitate analyses that will clarify potential benefit–risk differences between treatment regimens, including regimens incorporating one‐time gene replacement therapy. The relative prevalence of different regimens is also poorly understood. We sought to devise a rational, systematic, broadly applicable approach for defining and grouping *SMN*‐targeted DMT treatment scenarios that will facilitate future analyses aimed at exploring potential differences in clinical outcomes and address evidence gaps in the developing field of SMA DMTs. We also sought to describe the relative prevalence of various *SMN*‐targeted DMT scenarios observed in the RESTORE registry (NCT04174157), a prospective, multicenter, multinational, noninterventional, treatment‐neutral registry of patients with SMA, which was designed to integrate with existing real‐world data.^14^

## Methods

To classify various treatment scenarios, definitions were developed by a group of neuromuscular experts representing a broad geographic range and varied clinical practice settings. Once consensus was reached on treatment definitions, these definitions were applied to patients enrolled in the RESTORE registry^14^ (as of 20 December 2022) to examine the relative real‐world prevalence of each treatment strategy.

## Results

**Figure 1:**
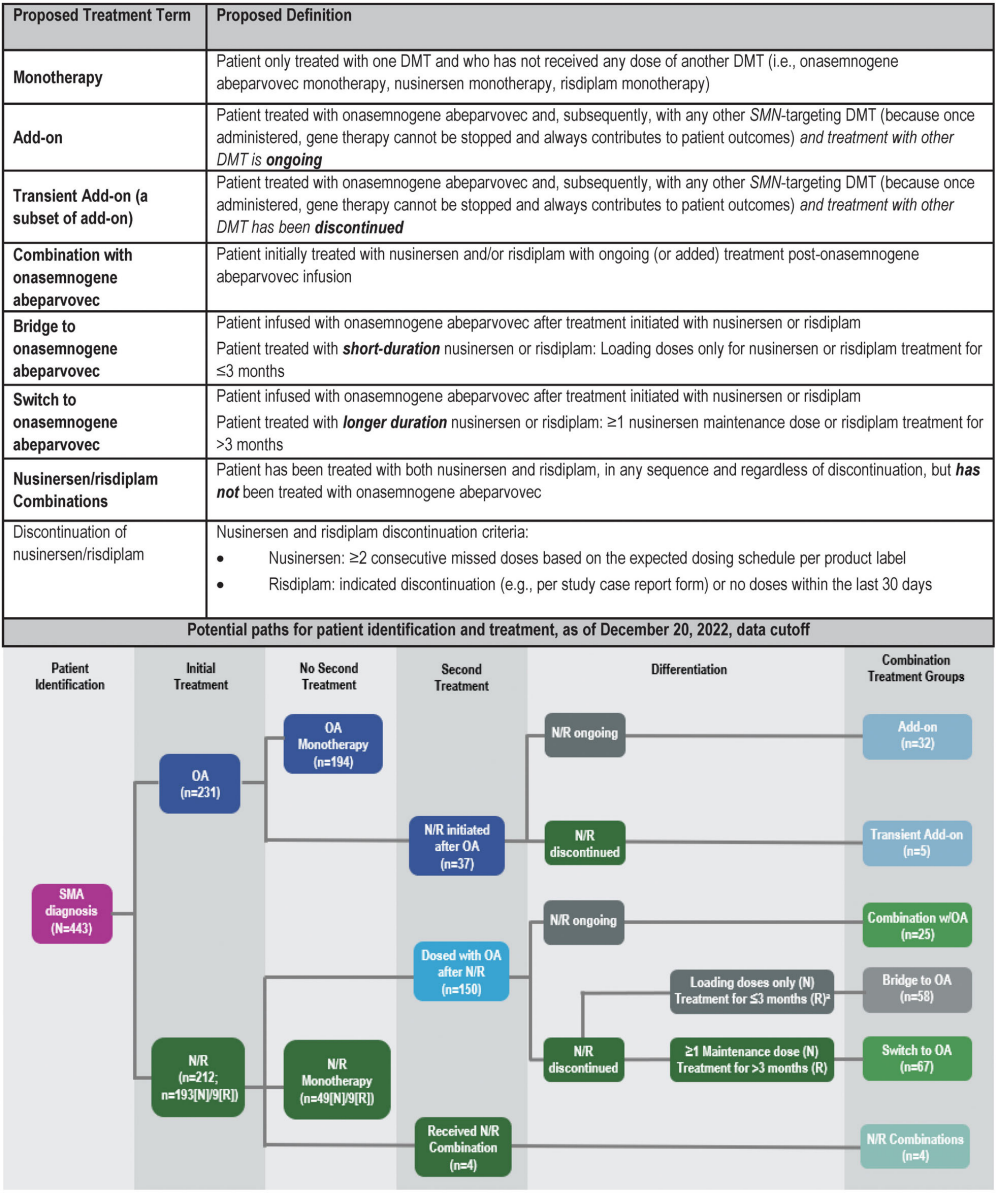
Proposed treatment terms and definitions and potential paths for patient identification and treatment. DMT, disease‐modifying treatment; N, nusinersen; OA, onasemnogene abeparvovec; R, risdiplam; SMA, spinal muscular atrophy. *SMN*, *survival motor neuron*. ^a^Nusinersen/risdiplam discontinued, ***or*** no doses of nusinersen/risdiplam received after onasemnogene abeparvovec infusion.

Our proposed treatment classification is primarily based on a two‐part differentiation: initial therapy received (gene therapy or *SMN2* splicing modifier) and persistence or discontinuation of subsequent DMT(s). This resulted in six polytherapy treatment categories: add‐on, transient add‐on, combination with onasemnogene abeparvovec, bridge to onasemnogene abeparvovec, switch to onasemnogene abeparvovec, and nusinersen/risdiplam combinations (Fig. 1). Because onasemnogene abeparvovec is a one‐time therapy that provides ongoing SMN protein expression, any treatment administered after infusion with onasemnogene abeparvovec is termed add‐on. Add‐on treatment to onasemnogene abeparvovec that is discontinued is termed transient. Combination treatment includes initial treatment with nusinersen and/or risdiplam with ongoing or added treatment after infusion with onasemnogene abeparvovec. Bridge therapy is short‐term treatment with nusinersen or risdiplam serving as a bridge to gene therapy with onasemnogene abeparvovec.^12^ Switching therapy is longer term treatment with nusinersen or risdiplam prior to receiving onasemnogene abeparvovec.^13^ Bridging and switching are distinguished based on duration of therapy with an initial *SMN2* splicing modifier before the use of gene therapy, with bridging a short‐duration (loading doses only for nusinersen or risdiplam treatment for ≤3 months) and switching a longer duration (one or more nusinersen maintenance dose or risdiplam treatment for >3 months). We defined discontinuation as two or more consecutive missed doses based on the expected dosing schedule (nusinersen) or no doses within the last 30 days (risdiplam). Durations for bridging and switching were adopted as reasonable surrogates for distinguishing these two treatment patterns in the absence of direct knowledge of caregiver/provider intent. Definitions for discontinuation were adopted based on previously published/presented analyses.^15^, ^16^ Importantly, the actual time on nusinersen therapy before discontinuation is variable depending on phase of treatment (loading doses or maintenance doses).

As we described the relative prevalence of the various proposed *SMN*‐targeted DMT treatment scenarios, RESTORE data were available for 443 patients at the 20 December 2022, data cutoff (Table 1; *see* Fig. 1). Of the patients in this analysis who initiated treatment with onasemnogene abeparvovec, 84.0% (*n* = 194/231) received monotherapy compared with 27.4% (58/212) of patients initiating treatment with either nusinersen or risdiplam (nusinersen monotherapy, *n* = 49; risdiplam monotherapy, *n* = 9). Of the 37 patients receiving add‐on DMT, treatment was ongoing at the time of data cutoff for 32 out of 37 patients (86.5%), with five patients receiving transient add‐on therapy. Of the 150 patients receiving onasemnogene abeparvovec after initial nusinersen or risdiplam, 38.7% (*n* = 58/150) received short‐term bridging therapy, 44.7% (67/150) received switching therapy, and 16.7% (*n* = 25/150) received combination with onasemnogene abeparvovec. Nusinersen/risdiplam combination therapy was uncommon in this patient sample, accounting for <1% of patients (*n* = 4).

**Table 1:** RESTORE treatment distribution.

Treatment	Patients, *n* (%)
All RESTORE patients, 20 December 2022, data cutoff	443 (100)
Monotherapy	
Onasemnogene abeparvovec monotherapy	194 (43.8)
Nusinersen monotherapy	49 (11.1)
Risdiplam monotherapy	9 (2.0)
Add‐on	32 (7.2)
Transient Add‐on	5 (1.1)
Combination with onasemnogene abeparvovec	25 (5.6)
Bridge to onasemnogene abeparvovec	58 (13.1)
Switch to onasemnogene abeparvovec	67 (15.1)
Nusinersen/risdiplam combinations	4 (0.9)

We also assessed patient demographics and characteristics associated with monotherapy and the proposed add‐on and combination *SMN*‐targeted DMT treatment scenarios (add‐on, transient add‐on, combination with onasemnogene abeparvovec, bridge to onasemnogene abeparvovec, and switch to onasemnogene abeparvovec) for patients from RESTORE who had received one‐time gene replacement therapy with onasemnogene abeparvovec (Table S1). The greatest percentage of patients in each treatment group had two *SMN2* copies (range: 44.4%–100%) and SMA type 1 (range: 33.3%–100%), except for patients receiving nusinersen monotherapy, with the greatest percentage of patients having three *SMN2* copies (61.2%) and SMA type 3 (47.0%). Overall, patient demographics and clinical characteristics were similar between groups for the patients receiving add‐on, transient add‐on, combinations with onasemnogene abeparvovec, bridging to onasemnogene abeparvovec, and switching to onasemnogene abeparvovec, but varied between the 187 patients in the treatment groups and the 194 patients receiving onasemnogene abeparvovec monotherapy. Approximately half of the patients receiving onasemnogene abeparvovec monotherapy (52.1%) were symptomatic at SMA diagnosis compared with a greater majority of patients in the add‐on and combination treatment groups (range: 75.9%–100%). Only 29.9% of patients in the groups receiving add‐on or combination *SMN*‐targeted DMT treatment scenarios (i.e., add‐on, transient add‐on, combinations with onasemnogene abeparvovec, bridging to onasemnogene abeparvovec, switching to onasemnogene abeparvovec) were identified by newborn screening (range: 22.4%–46.9%), with the greatest percentage of patients identified by newborn screening receiving onasemnogene abeparvovec monotherapy (58.3%). Despite having similar median age at symptom onset and first treatment, patients in groups receiving add‐on, transient add‐on, combinations with onasemnogene abeparvovec, bridging to onasemnogene abeparvovec, and switching to onasemnogene abeparvovec treatment scenarios were older at SMA diagnosis (median age 3 months) compared with patients receiving onasemnogene abeparvovec monotherapy (median age 1 month). The 187 patients in the groups receiving add‐on, transient add‐on, combinations with onasemnogene abeparvovec, bridging to onasemnogene abeparvovec, and switching to onasemnogene abeparvovec treatment scenarios were heavier (median weight 7.5 kg) and older (median age 9 months) compared with the 181 patients receiving onasemnogene abeparvovec monotherapy with weight data available (median weight 5.2 kg, median age 3 months). Most patients had a 1‐month interval between diagnosis and treatment, except for patients receiving nusinersen monotherapy (median interval, 2 months) and bridging to onasemnogene abeparvovec (median interval, 0 months).

## Discussion

Our description of the relative prevalence of the various *SMN*‐targeted DMT treatment scenarios observed in the RESTORE registry has some limitations. Although a global disease registry, RESTORE has primarily enrolled patients treated in the United States, and treatment patterns in other parts of the world may differ. In addition, treatment regimens that include only the two *SMN2* splicing modifiers (nusinersen and risdiplam) may be underrepresented, and our proposed classification does not differentiate between various potential combination scenarios involving only the two *SMN2* splicing modifiers (nusinersen and risdiplam). However, our classification system reflects the authors' collective expert opinion that the primary interest in differentiating treatment sequences or combinations for SMA lies with regimens that include onasemnogene abeparvovec because, unlike nusinersen or risdiplam, the gene therapy will theoretically continue to produce sufficient functional SMN protein over the lifetime of the patient.

Several potential treatment scenarios are encompassed within the “Combination with onasemnogene abeparvovec” group. Although it is possible that these various scenarios could be associated with important differences in clinical outcomes, multiple variables (e.g., age at diagnosis, duration of symptoms, timing of treatments, disease severity at treatment initiation) confound answering this question. Small patient numbers representing each specific (often unique) scenario preclude meaningful analysis of outcomes within this group. All treatment groups include infants identified at risk for SMA by newborn screening as well as clinically diagnosed patients, indicating a potentially significant degree of heterogeneity within each group that may complicate analysis of differential outcomes by treatment regimen. Alternative means of classifying and differentiating SMA combination treatment groups are possible. We believe, however, that our proposed system is broadly applicable and does not preclude stratification according to other criteria (e.g., according to patient age during combination therapy, or analyses that consider ongoing exposure to previously administered *SMN2* splicing modifiers [i.e., analyses that consider the half‐life of these treatments]) that may be more suitable to approach specific clinical questions. Our classification exercise was limited to *SMN*‐targeting treatments for SMA. Although clinical trials investigating new classes of SMA treatments (e.g., apitegromab, a selective myostatin inhibitor) are currently underway,^17^ present experience with these new treatments in real‐world practice is insufficient to inform rationale for integration into a classification schema such as this. Clinical experience with investigational myostatin inhibitors has thus far been limited to older patients with SMA (≥2 years of age), and the majority of the incident SMA population now receiving initial (and potentially additional) treatment are well below this age. Our classification schema could be expanded in the future to include non–*SMN*‐directed treatments, as well as other treatments currently under investigation for older SMA patients, such as intrathecal onasemnogene abeparvovec.

Although add‐on treatment was ongoing as of the data cutoff in the majority of cases, this percentage is certain to fluctuate over time. We expect that patient “migration” between treatment groups will be common as treatments are initiated and discontinued. In this cohort from RESTORE, the majority of patients who initiated treatment with either nusinersen or risdiplam continued on to receive onasemnogene abeparvovec. Future analyses will explore the rationales for add‐on and combination treatments, and alignment of stated rationale with patient status at the time of add‐on initiation. We will also explore clinical outcomes (including motor function assessments, motor milestone achievements, adverse events, and duration of additional treatment) for patients in the RESTORE registry according to these treatment definitions.

## Author Contributions

All authors contributed to the conception and design of this study. All authors contributed to analysis discussions, acquisition and analysis of data, and all authors reviewed and edited the manuscript, and approved its submission.

## Conflict of Interest

**CMP** is a site principal investigator for Astellas, Biogen, Catabasis, CSL Behring, Novartis Gene Therapies, Inc., Pfizer, PTC, Sarepta, and Scholar Rock clinical trials, and has received honoraria for advisory board participation from Biogen, Novartis Gene Therapies, Inc., Novartis, Roche, and Sarepta; and speaker's fees from Biogen and Novartis Gene Therapies, Inc. **EM** has received personal compensation for clinical trial consulting, serving on scientific advisory boards, and research funding from Novartis Gene Therapies, Inc. **RSF** has received personal compensation for consulting and for advisory board participation from Novartis Gene Therapies, Inc., Biogen, Novartis, Roche, and Scholar Rock; editorial fees from Elsevier for co‐editing a neurology textbook; license fees from the Children's Hospital of Philadelphia; and research funding from Novartis Gene Therapies, Inc., Biogen, Roche/Genentech, and Scholar Rock. **JK** has received honoraria for clinical research and/or consultancy activities from Biogen, Novartis Gene Therapies, Inc., Pfizer, Roche, Sarepta and Scholar Rock. **DCD** has received personal compensation for consulting for advisory board participation from Novartis Gene Therapies, Inc., Biogen, Cytokinetics, Ionis Pharmaceuticals, Inc., Mallinckrodt, Metafora, PTC Therapeutics, Roche, Sanofi, Sarepta Therapeutics, and Ultragenyx, and the n‐lorem Foundation and SMA Foundation. He has no financial interests in these companies. He has received compensation as a consultant from Novartis Gene Therapies, Inc., Biogen, and Sarepta Therapeutics. He has received research grants from the Hope for Children Research Foundation, National Institutes of Health, SMA Foundation, Cure SMA, GliaPharm, Rocket Pharma, Glut1 Deficiency Foundation, and the US Department of Defense; he also has received clinical trial funding from Biogen, Mallinckrodt, PTC Therapeutics, Sarepta Therapeutics, Scholar Rock, and Ultragenyx; and serves as a member of the DSMB for Aspa Therapeutics. **FM** has received honoraria for scientific advisory board participation from Novartis Gene Therapies, Inc., Biogen, Novartis, Lilly, PTC, Roche, Dyne Therapeutics and Sarepta, and grants and personal fees from Novartis Gene Therapies, Inc., Biogen, and Roche. **KS** is a site principal investigator for Biogen, Novartis Gene Therapies, Inc., and Chugai/Roche clinical trials; has served on advisory boards for Novartis Gene Therapies, Inc./Novartis, Biogen, and Chugai/Roche; and has received speaker's fees from Biogen, Novartis, and Chugai/Roche. **EFT** has received personal compensation for consultancy from Novartis Gene Therapies, Inc., Biogen, Biologiz, Cytokinetics, Novartis, and Roche, and research funding from Biogen/Ionis and Roche. **ID** has received personal compensation for lectures and/or scientific board meetings from Novartis Gene Therapies, Inc., Biogen, BioMarin, PTC Therapeutics, and Sarepta. **SQ‐R** is a site principal investigator for clinical trials of Biogen and Novartis Gene Therapies, Inc.; has served as a consultant and has participated on advisory boards for Novartis Gene Therapies, Inc., Biogen, and Roche; and has received travel and speaker honoraria from Biogen, Novartis, and Roche. **KB**, **DR**, and **EF** are employees of Novartis Gene Therapies and own stock/other equities. **LS** has received personal compensation from Novartis Gene Therapies, Inc., Biogen, Inc., Biophytis, Cytokinetics, Dynacure, Roche, Santhera, and Sarepta Therapeutics, and research funding from Novartis Gene Therapies, Inc., Biogen, Dynacure, and Roche.

## Supporting information


**Table S1**.
