# Supplementary material for: Combination disease‐modifying treatment in spinal muscular atrophy: A proposed classification
Source: Ann Clin Transl Neurol. 2023 Sep 10;10(11):2155–60. doi: 10.1002/acn3.51889 (PMC10646995; doi:10.1002/acn3.51889)
Supplement: Supplementary file 1 — Table S1. [file ACN3-10-2155-s001.docx]

**Supplemental Materials**

**Table S1. Patient demographics/characteristics by treatment distribution**

| **Patients, n (%)** | | | | | | | | | |
| --- | --- | --- | --- | --- | --- | --- | --- | --- | --- |
| **Demographics/**  **characteristics** | **Monotherapy** | | | **Add-on and Combination Treatment Scenarios** | | | | | **All Patients**  **in the**  **Add-on and Combination Treatment Scenarios**  **(n=187)** |
|  | **Onasemnogene Abeparvovec**  **(n=194)** | **Nusinersen**  **(n=49)** | **Risdiplam**  **(n=9)** | **Add-on (n=32)** | **Transient Add-on (n=5)** | **Combination with Onasemnogene Abeparvovec (n=25)** | **Bridge to Onasemnogene Abeparvovec (n=58)** | **Switch to Onasemnogene Abeparvovec (n=67)** |  |
| **Sex, n (%)** | | | | | | | | | |
| Male | 92 (47.4) | 24 (49.0) | 5 (55.6) | 11 (34.4) | 3 (60.0) | 10 (40.0) | 24 (41.4) | 32 (47.8) | 80 (42.7) |
| Female | 102 (52.6) | 25 (51.0) | 4 (44.4) | 21 (65.6) | 2 (40.0) | 15 (60.0) | 34 (58.6) | 35 (52.2) | 107 (57.2) |
| **Country, n (%)** | | | | | | | | | |
| United States | 153 (78.9) | 34 (69.4) | 8 (88.9) | 29 (90.7) | 5 (100.0) | 23 (92.0) | 33 (56.9) | 34 (50.8) | 124 (66.3) |
| Greece | 4 (2.1) | 4 (8.2) | 0 (0) | 0 (0) | 0 (0) | 0 (0) | 0 (0) | 3 (4.5) | 3 (1.6) |
| Ireland | 0 (0) | 2 (4.1) | 0 (0) | 0 (0) | 0 (0) | 0 (0) | 1 (1.7) | 4 (6.0) | 5 (2.7) |
| Israel | 3 (1.6) | 0 (0) | 0 (0) | 0 (0) | 0 (0) | 0 (0) | 1 (1.7) | 2 (3.0) | 3 (1.6) |
| Japan | 20 (10.3) | 3 (6.1) | 0 (0) | 1 (3.1) | 0 (0) | 2 (8.0) | 20 (34.5) | 13 (19.4) | 36 (19.3) |
| Portugal | 5 (2.6) | 2 (4.1) | 0 (0) | 0 (0) | 0 (0) | 0 (0) | 3 (5.2) | 3 (4.5) | 6 (3.2) |
| Romania | 0 (0) | 1 (2.0) | 0 (0) | 1 (3.1) | 0 (0) | 0 (0) | 0 (0) | 0 (0) | 1 (0.5) |
| Russia | 5 (2.6) | 1 (2.0) | 1 (11.1) | 0 (0) | 0 (0) | 0 (0) | 0 (0) | 4 (6.0) | 4 (2.1) |
| South Korea | 0 (0) | 0 (0) | 0 (0) | 0 (0) | 0 (0) | 0 (0) | 0 (0) | 2 (3.0) | 2 (1.1) |
| Taiwan | 4 (2.1) | 2 (4.1) | 0 (0) | 1 (3.1) | 0 (0) | 0 (0) | 0 (0) | 2 (3.0) | 3 (1.6) |
| **Number of *SMN2* copies, n (%)** | | | | | | | | | |
| One | 4 (2.1) | 0 (0) | 0 (0) | 0 (0) | 0 (0) | 1 (4.0) | 0 (0) | 0 (0) | 1 (0.5) |
| Two | 91 (46.9) | 8 (16.3) | 4 (44.5) | 26 (81.3) | 5 (100.0) | 23 (92.0) | 39 (67.2) | 51 (76.1) | 144 (77.1) |
| Three | 79 (40.7) | 30 (61.2) | 3 (33.3) | 6 (18.8) | 0 (0) | 1 (4.0) | 17 (29.3) | 16 (23.9) | 40 (21.4) |
| Four | 12 (6.2) | 10 (20.4) | 2 (22.2) | 0 (0) | 0 (0) | 0 (0) | 1 (1.7) | 0 (0) | 1 (0.5) |
| More than four | 7 (3.6) | 1 (2.1) | 0 (0) | 0 (0) | 0 (0) | 0 (0) | 1 (1.7) | 0 (0) | 1 (0.5) |
| Missing | 1 (0.5) | 0 (0) | 0 (0) | 0 (0) | 0 (0) | 0 (0) | 0 (0) | 0 (0) | 0 (0) |
| **SMA type, n (%)** | | | | | | | | | |
| 0 | 0 (0) | 0 (0) | 0 (0) | 0 (0) | 0 (0) | 1 (4.0) | 0 (0) | 0 (0) | 1 (0.5) |
| 1 | 85 (43.8) | 8 (16.3) | 3 (33.3) | 27 (84.4) | 5 (100.0) | 23 (92.0) | 35 (60.3) | 54 (80.6) | 144 (77.0) |
| 2 | 25 (12.9) | 13 (26.5) | 2 (22.2) | 3 (9.4) | 0 (0) | 1 (4.0) | 9 (15.5) | 7 (10.5) | 20 (10.7) |
| 3 | 4 (2.1) | 23 (47.0) | 1 (11.1) | 2 (6.3) | 0 (0) | 0 (0) | 0 (0) | 0 (0) | 2 (1.1) |
| Missing | 80 (41.2) | 5 (10.2) | 3 (33.3) | 0 (0) | 0 (0) | 0 (0) | 14 (24.1) | 6 (9.0) | 20 (10.7) |
| **Identified by newborn screening, n (%)** | 113 (58.3) | 7 (14.2) | 0 (0) | 15 (46.9) | 2 (40.0) | 7 (28.0) | 17 (29.3) | 15 (22.4) | 56 (29.9) |
| **Symptomatic at diagnosis, n (%)** | 101 (52.1) | 44 (89.8) | 7 (77.8) | 26 (81.3) | 4 (80.0) | 25 (100.0) | 44 (75.9) | 58 (87.6) | 157 (83.9) |
| **Age at initial SMA diagnosis, months** | | | | | | | | | |
| Median (range) | 1 (0, 27) | 32 (0, 502) | 14 (3, 105) | 2 (0, 22) | 5 (0, 5) | 2 (0, 17) | 2.5 (0, 22) | 5 (0, 23) | 3 (0, 23) |
| IQR | 0–6 | 9–78 | 8–34 | 0–5 | 4–5 | 1–4 | 0–8 | 1–8 | 1–7 |
| Mean (SD) | 4.1 (5.98) | 68.3 (96.9) | 27.1 (31.7) | 4.13 (6.1) | 3.8 (2.2) | 3.52 (4.2) | 4.98 (5.8) | 5.66 (5.4) | 4.8 (5.4) |
| **Age at symptom onset, months** | | | | | | | | | |
| Median (range) | 2 (0, 23) | 15 (0, 168) | 5 (0, 36) | 2 (0, 14) | 2 (0, 5) | 1 (0, 8) | 2 (0, 10) | 2 (0, 9) | 2 (0, 14) |
| IQR | 1–6 | 6–36 | 3–12 | 0–3 | 1–3 | 1–3 | 0–4 | 1–5 | 0–4 |
| Mean (SD) | 4.3 (4.9) | 29.7 (39.3) | 10.4 (12.1) | 2.0 (2.7) | 2.2 (1.9) | 2.2 (2.5) | 2.7 (3.2) | 2.6 (2.2) | 2.4 (2.6) |
| Missing, n | 69 | 5 | 2 | 0 | 0 | 0 | 14 | 5 | 19 |
| **Age at first treatment, months** | | | | | | | | | |
| Median (range) | 3 (0, 54) | 37 (0, 507) | 23 (3, 106) | 3 (0, 23) | 6 (1, 7) | 3 (0, 17) | 3 (0, 22) | 6 (0, 21) | 3 (0, 23) |
| IQR | 1–9 | 13–81 | 8–34 | 1.5–7 | 6–6 | 2–5 | 2–8 | 2–13 | 2–9 |
| Mean (SD) | 6.5 (8.4) | 72.8 (97.9) | 29.2 (31.4) | 6.1 (6.6) | 5.2 (2.3) | 4.2 (3.8) | 5.5 (5.8) | 7.6 (6.1) | 6.2 (5.9) |
| **Interval between diagnosis and treatment, months** | | | | | | | | | |
| Median (range) | 1 (0, 48) | 2 (0, 37) | 1 (0, 9) | 1 (0, 16) | 1 (1, 2) | 1 (0, 3) | 0 (0, 3) | 1 (0, 20) | 1 (0, 20) |
| IQR | 1–2 | 1–4 | 0–2 | 1–1.5 | 1–2 | 0–1 | 0–1 | 0–2 | 0–1 |
| Mean (SD) | 2.4 (6.2) | 4.5 (7.2) | 2.1 (3.2) | 2.0 (3.5) | 1.4 (0.5) | 0.7 (0.7) | 0.6 (0.8) | 2.2 (3.8) | 1.4 (2.8) |
| **Weight at onasemnogene abeparvovec administration, kg** | | | | | | | | | |
| Median (Min, Max) | 5.2 (1.6, 12.5) | N/A | N/A | 5.7 (3.2, 12.2) | 5.8 (4.1, 7) | 8.0 (2.6, 12.0) | 7.1 (3.1, 11.9) | 8.5 (4.2, 15.0) | 7.5 (2.6, 15) |
| IQR | 4–7.8 | N/A | N/A | 4.5–7.8 | 5.1–6.3 | 6.3–9.7 | 5.9–8.75 | 7.3–9.65 | (6.1–9.2) |
| Mean (SD) | 5.9 (2.3) | N/A | N/A | 6.4 (2.4) | 5.7 (1.1) | 8.0 (2.2) | 7.4 (1.9) | 8.4 (1.9) | 7.6 (2.2) |
| Missing, n | 13 | N/A | N/A | 3 | 0 | 0 | 6 | 7 | 16 |
| **Age at onasemnogene abeparvovec administration, months** | | | | | | | | | |
| Median (Min, Max) | 3 (0, 54) | N/A | N/A | 3 (0, 23) | 6 (1, 7) | 9 (1, 31) | 7 (1, 45) | 19 (2, 38) | 9 (0, 45) |
| IQR | 1–9 | N/A | N/A | 1.5–7 | 6–6 | 5–18 | 5–13 | 12–23 | 5–20 |
| Mean (SD) | 6.5 (8.4) | N/A | N/A | 6.1 (6.6) | 5.2 (2.4) | 12.0 (8.4) | 9.7 (7.7) | 17.7 (7.5) | 12.1 (8.7) |
| **Duration of follow up after onasemnogene abeparvovec administration, months** | | | | | | | | | |
| Median (range) | 14.98 (0.03, 49.2) | N/A | N/A | 25.9 (13.3, 40.2) | 29.0 (21.1, 34.7) | 18.3 (0.9, 46.6) | 20.8 (0.4, 44.6) | 23.6 (0.3, 39.6) | 23.0 (0.3, 46.6) |
| IQR | 7–23.8 | N/A | N/A | 19.5–32.1 | 25.7–31.1 | 11.7–35.4 | 13.1–25.8 | 15.3–28.4 | 15.3–29.3 |
| Mean (SD) | 15.8 (10.3) | N/A | N/A | 26.5 (7.8) | 28.3 (5.2) | 22.8 (14.2) | 19.9 (10.1) | 21.5 (9.7) | 22.2 (10.4) |

IQR, interquartile range; N/A, not applicable; SD, standard deviation; SMA, spinal muscular atrophy; *SMN2*, *survival motor neuron 2* gene.
